# Guided Self-Help Cognitive Behavioural Therapy for Depression in Primary Care: A Randomised Controlled Trial

**DOI:** 10.1371/journal.pone.0052735

**Published:** 2013-01-11

**Authors:** Christopher Williams, Philip Wilson, Jill Morrison, Alex McMahon, Walker Andrew, Lesley Allan, Alex McConnachie, Yvonne McNeill, Louise Tansey

**Affiliations:** 1 Institute of Health and Wellbeing, University of Glasgow, Strathclyde, Scotland, United Kingdom; 2 Dental School, University of Glasgow, Strathclyde, Scotland, United Kingdom; 3 Robertson Centre, University of Glasgow, Strathclyde, Scotland, United Kingdom; Linkoping University, Sweden

## Abstract

**Background:**

Access to Cognitive behavioural therapy (CBT) for depression is limited. One solution is CBT self-help books.

*Trial Objectives*: To assess the impact of a guided self-help CBT book (GSH-CBT) on mood, compared to treatment as usual (TAU).

Hypotheses:

1. GSH-CBT will have improved mood and knowledge of the causes and treatment of depression compared to the control receiving TAU
2. Guided self-help will be acceptable to patients and staff.

**Methods and Findings:**

*Participants*: Adults attending seven general practices in Glasgow, UK with a BDI-II score of ≥14. 141 randomised to GSH-CBT and 140 to TAU.

*Interventions*: RCT comparing ‘*Overcoming Depression: A Five Areas Approach*’ book plus 3–4 short face to face support appointments totalling up to 2 hours of guided support, compared with general practitioner TAU.

*Primary outcome*: The BDI (II) score at 4 months.

*Numbers analysed*: 281 at baseline, 203 at 4 months (primary outcome), 117 at 12 months.

*Outcome*: Mean BDI-II scores were lower in the GSH-CBT group at 4 months by 5.3 points (2.6 to 7.9, p<0.001). At 4 and 12 months there were also significantly higher proportions of participants achieving a 50% reduction in BDI-II in the GSH-CBT arm. The mean support was 2 sessions with 42.7 minutes for session 1, 41.4 minutes for session 2 and 40.2 minutes of support for session 3.

*Adverse effects/Harms*: Significantly less deterioration in mood in GSH-CBT (2.0% compared to 9.8% in the TAU group for BDI—II category change).

**Limitations:**

*Weaknesses*: Our follow-up rate of 72.2% at 4 months is better than predicted but is poorer at 12 months (41.6%). In the GSH-CBT arm, around 50% of people attended 2 or fewer sessions. 22% failed to take up treatment.

**Conclusions:**

GSH-CBT is substantially more effective than TAU.

**Trial Registration:**

Controlled-Trials.com ISRCTN13475030

## Introduction

Cognitive behavioural therapy (CBT) is a short-term psychological therapy that is effective in the treatment of depression [1]. CBT is usually provided by specialist psychotherapists; but access to such services is generally limited. One possible solution is to use self-help materials incorporating a CBT approach, or ‘bibliotherapy’ [2].

Self-help is viewed very positively by the public [3]. A recent systematic analysis has confirmed the overall effectiveness of CBT self-help and identified that packages are best delivered as guided self-help (GSH), with guidance/support from a worker who does not necessarily have to be clinically qualified [4]. This gives greater improvement for depression than unguided/unsupported self-help.

*Overcoming Depression: A Five Areas Approach'* [5] is a structured self-help treatment for depression. It contains stand alone CBT workbooks covering topics such as Practical Problem Solving, Being Assertive, Using Antidepressant Medication, Overcoming Sleep Problems and others. The content was developed in liaison with primary and secondary health care practitioners [6]. Workbooks are designed to be jargon-free and have a low reading age, high accessibility [7] and can be used in a modular fashion. One model of time-limited and focused delivery of GSH-CBT is the use of a “2+1” design comprising two short support sessions one week apart followed by a third session at a later date [8].

We aimed to evaluate the use of the workbooks supported by a non-clinically qualified psychology graduate using three 40 minute appointments at 1, 2 and 4 weeks (the 2+1 model) alongside standard care by the family physician (GP), with GP treatment as usual (TAU) alone.

The study hypotheses were:

1. Patients using GSH-CBT will have:
  - Improved mood measured by the Beck Depression Inventory (BDI-II) [9] at 4 months
  - Improvement in the Clinical Outcomes in Routine Evaluation - Outcome Measure (CORE-OM) [10]
  - Improved knowledge of the causes and treatment of depression compared to the control group receiving treatment as usual
2. Written self-help will be acceptable to both patients and staff within a primary care setting.

## Methods

The protocol for this trial and supporting CONSORT checklist are available as supporting information; see Checklist S1 and Protocol S1. Ethics approval was granted by NHS Glasgow Primary Care – Community and Mental Health Research Ethics Committee - 02/24.

### Patients

Patients aged 18 years or over presenting to one of seven National Health Service general practices in Glasgow, Scotland, UK with symptoms of depression, a Beck Depression Inventory–II (BDI-II) score of 14 or more, and ability to use the written materials (i.e. no visual or reading problems, learning difficulties or dementia) were offered entry into the study. Any clinical member of the primary care team (general practitioners - GPs - or nurses) could refer to the study. In order to exclude potential participants for whom the intervention might be clinically inappropriate in a consistent way, a simple exclusion algorithm based on the BDI-II scoring was adopted using cut-off points chosen by the authors. Patients with active suicidal intent (scoring 2 or more on the BDI-II suicidal thoughts item) were excluded as such patients need more than GCBT-SH or TAU. Also excluded were those who were unable to use the materials because of impaired concentration and motivation (scoring 7 or more on the combined BDI-II items for energy, concentration difficulty and tiredness – items 15, 19 and 20) as they would experience difficulty in using the written materials effectively.

### Study Design

This is a parallel group randomised controlled trial (RCT) comparing GSH-CBT plus routine primary care treatment with primary care treatment as usual alone (TAU). Allocation was at an individual level. Any apparently suitable patient with low mood expressing an interest in the study were seen by the research assistant (RA) within a week at their general practice.

At the initial appointment, the RA checked the inclusion/exclusion criteria and obtained written informed consent from all participants. Baseline assessment measures were completed with the RA (CORE-OM; and Patient Questionnaire – PQ [11] addressing mental health literacy. The PQ questionnaire is a non-validated questionnaire asking about the participants previous and current attitudes and use of self-help resources, their knowledge of the causes and treatment options for depression, and self-rated knowledge in identifying and changing problems such as negative thoughts (Mental health literacy). The CORE-OM comprises 34 items and measures four domains (subjective well-being, symptoms, life-functioning, and risk to self and to others). Each item is scored on a 5-point scale ranging from 0 (not at all) to 4 (most or all of the time). We report here only CORE total score.

After baseline measures, patients were randomised using an automated remote telephone system to the GSH-CBT or TAU groups. The referring clinician was informed about whether patients were eligible, whether they entered the study and of their score on the BDI-II suicide screening question.

If participants were allocated to the GSH-CBT intervention, they were offered their first appointment with the psychology graduate within 7 days at their own general practice. Three face to face support sessions of approximately 40 minutes could be delivered per protocol; with the option of a fourth session if needed with a support/guidance target ranging from 0–160 minutes depending on take-up. The first appointment focussed on an introduction to the use of the self-help materials. The patient was given a copy of Workbook 1 (“Understanding depression”) and instructed on how to use it. At session 2, the first workbook was reviewed before a joint decision identified an additional 1–2 treatment workbooks to be used between sessions 2 and 3. These were chosen on the basis of the initial self-assessment in the Understanding depression workbook. At session 3, there was a final review of their progress. The relapse prevention workbook and up to one or two additional workbooks were also offered at this final appointment. The workbooks aimed to be accessible with a reading age of around 12 years, and aimed to communicate key CBT principles in a low jargon way. Case examples, illustrations, text and interactive worksheets encouraged users to self-assess, and then choose which topics (workbooks) they would work on. Each workbook included a Putting into Practice (homework) plan to encourage application in the reader's own life. The choice of workbooks followed a core/options approach where the initial workbook (self-assessment) helped identify what problem areas the person wished to work on. In the final support session the focus was on the Planning for the Future (relapse prevention) workbook. At any time during treatment, patients could arrange to see their doctor or other health care practitioner as normal. The intervention by the psychology graduate was only to support the use of the self-help materials using a written support protocol and “advice” separate from the intervention was not offered. The GP was informed that the participant had been seen and discharged at the end of GCBT-SH support.

The support protocol focused on using and applying one to two workbooks per week. The support worker encouraged the participant to read, answer questions and plan how to put what was being learned into practice. Each session allowed progress or barriers to progress to be reviewed and plans to overcome these barriers to be discussed. The support worker was a non-clinically qualified psychology graduate with a basic honours degree in undergraduate psychology. During the course of the project only one support worker was used at any one time, and three support workers delivered the intervention over the course of the project. Face to face supervision was provided on a weekly or fortnightly basis.

The study methodology was approved by the local research ethics committee (NHS Glasgow Primary Care – Community and Mental Health Research Ethics Committee - 02/24), and the trial registered (ISRCTN13475030). The sponsor was NHS Greater Glasgow and Clyde.

### Outcome measures

In both arms, measures were obtained at baseline, and by mail at four and 12 months (see fig. S1). The primary outcome was a comparison between the BDI-II scores for the two randomised groups at 4 months. The BDI-II [9] is a 21 item self-rated questionnaire for depression, with each item rated 0–3 (score 0–63). A score of 0–13 is classified as minimal depression, 14–19 mild, 20–28 moderate and 29–63 severe depression).

Psychological symptoms and social functioning (using the CORE-OM [10] scale), and acceptability of the intervention (using the Client Satisfaction Questionnaire- CSQ) [12] were compared.

### Statistical Analysis

The sample size in this study is based on a published comparison between cognitive behavioural therapy and usual care using the BDI-II score at 4 months [13]. That study found a between groups effect size of 0.43 for their CBT intervention. This reflects an improvement of 4.5 points on the BDI-II – a clinically useful gain which is in the usual range selected by similar studies [14]. The study experienced a drop out rate of 10% by the 4 month follow up. We had estimated a drop-out rate of 33% because of the lower level of therapist contact in our design. To achieve 85% power to detect a between-group difference of 0.43 standard deviations requires 99 participants per group, based on standard formulae for a two-sample t-test. To allow for loss to follow-up, we aimed to randomise 300 patients. Given the slightly lower rate of loss to follow-up at 4 months of 28%, recruitment ceased at 281 subjects, and 4-month follow-up was achieved for 203 randomised participants.

All analyses were performed under the intention to treat principle, i.e. between-group comparisons were in relation to the randomised groups, regardless of participation with study interventions. Continuous outcome measures (BDI-II score, CORE-OM total score at 4 and 12 months, and measures of health literacy at 1 month) were compared between treatment groups at each relevant time point using analysis of covariance (ANCOVA), i.e. baseline-adjusted linear regression analysis. Estimates of mean differences between groups are reported with a 95% confidence interval (CI) and p-value. Analyses were initially carried out using only those subjects with available data. Additional analyses of BDI-II and CORE-OM total score were conducted after imputing missing values as equal to the baseline value.

For BDI-II scores, additional analyses were carried out of whether each individual achieved a 50% reduction in BDI-II score, relative to baseline. For these analyses, those with missing data are assumed not to have changed since baseline. Between-group differences are reported as an odds ratio with 95% CI and p-value.

Analyses were conducted using S-Plus for Windows v8.1. No adjustments were made for multiple comparisons. For the primary analysis, statistical significance was taken as 5%; for secondary analyses, p-values less than 5% were taken as indicative of true associations, with smaller p-values representing greater levels of evidence.

## Results

The flow of patients through the study is summarised in the Consort diagram in Figure S1.

### Baseline Clinical and Demographic Characteristics

Overall, 281 people entered the study – with 140 randomised to treatment as usual (TAU) and 141 to GSH-CBT. 94% of referrals came from the General practitioner, 5.7% from a practice nurse and 0.4% from a health visitor.

Characteristics of participants are summarised in Table 1.

**Table 1:** Characteristics of study participants and non-participants.

		Non- participants	Participants	
			TAU	GSH-CBT
N		100	140	141
Age (years)	Mean (SD)	44.0 (16.5)	43.1 (14.2)	40.4 (13.9)
Gender	N (%) Male	32 (32%)	51 (36.4%)	38 (27.0%)
	N (%) Female	68 (68%)	89 (63.6%)	103 (73.0%)
Current/recent use of self-help materials	N (%) Yes		11 (7.9%)	14 (9.9%)
	N (%) No		129 (92.1%)	127 (90.1%)
Current/recent antidepressants	N (%) Yes		87 (62.1%)	77 (54.6%)
	N (%) No		53 (37.9%)	64 (45.4%)
Currently working/employed	N (%) Yes		103 (73.6%)	99 (70.2%)
	N (%) No		15 (10.7%)	19 (13.5%)
	N (%) Not Known		22 (15.7%)	23 (16.3%)
Deprivation score^a^	Mean (SD)	5.2 (2.14)	4.23 (2.18)	4.38 (2.21)

a Carstairs Depcat [16], [17]: 1–7: 1 = most affluent areas; 7 = most deprived areas.

The Carstairs index, available for each postcode/zip code area of Scotland was used as a measure of social deprivation [15], [16]. Of patients who were screened for the study and were not recruited (n = 91), 21 were not interested in the study, 12 were not depressed enough (a score of <14 on the BDI-II), 24 were unable to use the materials and 34 were excluded based on the remaining exclusion criteria (low energy, concentration and motivation - 24, (24%), active suicidality - 10, (10%)).

The proportion using antidepressants at baseline in the TAU arm was 87/140 (62.1%) compared to 77/141 (54.6%) in GSH-CBT (Chi p = 0.246). There was no evidence of a significant difference in proportion of people taking antidepressants at baseline (TAU 87/140, GCBT-SH 77/141 p = 0.246), at 4 months (TAU 62/126, GCBT-SH 58/123 p = 0.844), and at 12 months (TAU 53/111, GCBT-SH 54/105 p = 0.686). In the TAU arm 28 stopped antidepressants and 11 started them between baseline and 4 months (p = 0.009). In the same time period, 21 stopped antidepressants and 13 started them in the GCBT-SH arm (p = 0.229). Between 4 months and 12 months, in the TAU arm 10 stopped and 9 started antidepressants (p = 1), and 10 stopped and 12 started antidepressants in the GCBT-SH arm (p = 0.83). There was therefore no evidence of a significant change in proportion of people taking antidepressants between baseline and 4 months, baseline and 12 months, and between the proportion taking antidepressants at 4 and 12 months in the logistic regression.

### Treatment

*Attendance and length of sessions in the GP-TAU arm (n = 140):* all patients had access to standard treatment from their family doctor (n = 140). This would usually entail monitoring, antidepressant prescription and referral for specialist psychological therapies as recommended by national treatment guidelines [1]. These were delivered within a National Health Service (NHS) setting in which access to care is free at the point of contact. Typically reviews would be weekly to monthly.

*Attendance and length of sessions in the guided self-help CBT arm (n = 141):* Overall, 45% (64) of patients attended all three sessions. 17% (14) attended two, 10% (14) one and 22% (32) failed to attend a single session. Only 5% (7) of participants required a fourth session. The mean number of sessions was 2 (sd 1.29). The mean time spent with the psychology assistant was 42.7 minutes for session 1, 41.4 minutes for session 2 and 40.2 minutes for session 3 respectively (times available for session 1 n = 83, session 2 n = 69 and session 3 n = 52).

Adherence to protocol was examined through regular supervision and through recording sessions. All recordings were examined and rated satisfactory against a brief adherence checklist [17].

### Rates of Discontinuation and Adverse Events

One person died during the course of the study (from the TAU arm) of an unrelated condition (non-Hodgkins lymphoma). Deterioration using the BDI-II score at month 4 compared to baseline was 25.5% (TAU) and 4.0% (GCBT-SH), and at 12 months 25.5% (TAU) and 8.2% (GCBT-SH). Other adverse events were recorded using the follow-up PQ questionnaire which addressed participant attitudes towards the guided self-help approach as well as re-testing mental health literacy [11].

### Efficacy

Results for all evaluable data are summarised in Table 2. The primary outcome data at four months were available for 203 patients (72.2%) with similar rates of follow-up in each arm. Mean BDI-II scores fell from 29.1 to 22.0 (TAU) and from 29.8 to 16.4 for the GSH-CBT arm (p<0.001). A lower response rate was obtained at 12 months (117/281, 41.6%). Table 3 shows sensitivity analyses where missing data have been imputed as the baseline value. CORE total scores were on average 0.26 points lower in the GSH-CBT group at four months (0.10 to 0.42, p = 0.002). Significant differences were also observed at 12 months and although somewhat smaller in size were still evident when imputing missing values as return to baseline.

**Table 2:** Outcomes at baseline, 4 months and 12 months in treatment as usual (TAU) and guided self-help CBT (GSH-CBT) groups, with treatment effect difference (TAU - GSH-CBT), 95% confidence interval (CI) and p-value, from ANCOVA analysis.

Outcome	Treatment	Baseline	4 Months		12 Months	
		N	N	Difference	N	Difference
		Mean (SD)	Mean (SD)	(95% CI)p-value	Mean (SD)	(95% CI)p-value
BDI-II	TAU	140	102	5.26 (2.60, 7.92)p<0.001	55	5.44 (1.27, 9.62)p = 0.012
		29.1 (9.2)	22.0 (12.2)		20.2 (14.0)	
	GSH-CBT	141	101		62	
		29.8 (9.6)	16.4 (11.1)		14.6 (11.2)	
CORE OM Total	TAU	140	110	0.26 (0.10, 0.42)p = 0.002	56	0.38 (0.14, 0.61)p = 0.002
		1.87 (0.60)	1.51 (0.77)		1.40 (0.85)	
	GSH-CBT	141	109		61	
		1.95 (0.59)	1.27 (0.74)		1.00 (0.70)	

**Table 3:** Outcomes (with missing data imputed as the baseline value) at baseline, 4 months and 12 months in treatment as usual (TAU) and guided self-help CBT (GSH-CBT) groups, with treatment effect difference (TAU - GSH-CBT), 95% confidence interval (CI) and p-value, from ANCOVA analysis.

Outcome	Treatment	Baseline	4 Months		12 Months	
		N	N	Difference	N	Difference
		Mean (SD)	Mean (SD)	(95% CI)p-value	Mean (SD)	(95% CI)p-value
BDI-II	TAU	140	140	3.51 (1.29, 5.74)p = 0.002	140	2.68 (0.46, 4.89)p = 0.018
		29.1 (9.2)	24.0 (11.9)		26.0 (12.2)	
	GSH-CBT	141	141		141	
		29.8 (9.6)	21.1 (13.3)		23.9 (13.4)	
CORE OM Total	TAU	140	140	0.19 (0.05, 0.33)p = 0.008	140	0.24 (0.10, 0.39)p = 0.001
		1.87 (0.60)	1.57 (0.75)		1.57 (0.77)	
	GSH-CBT	141	141		141	
		1.95 (0.59)	1.45 (0.78)		1.39 (0.82)	

Results of mental health literacy changes are summarised in Table 4.

**Table 4:** Self-Perceived Mental Health Literacy Scores (1 = very poor, 7 = excellent) at baseline and 1 month (evaluable data) in treatment as usual (TAU) and guided self-help CBT (GSH-CBT) groups, with treatment effect difference (TAU - GSH-CBT), 95% confidence interval (CI) and p-value, from ANCOVA analysis.

Outcome	Treatment	Baseline	1 Month	
		N	N	Mean Difference
		Mean (SD)	Mean (SD)	(95% CI)p-value
Overall knowledge of depression				
	TAU	132	55	−1.58 (−1.98, −1.18)p<0.001
		3.45 (1.65)	3.60 (1.36)	
	GSH-CBT	133	63	
		3.73 (1.56)	5.30 (1.07)	
Knowledge of the causes of depression				
	TAU	130	56	−1.77 (−2.24, −1.30)p<0.001
		3.19 (1.59)	3.36 (1.38)	
	GSH-CBT	133	63	
		3.57 (1.50)	5.33 (1.27)	
Ability to describe how depression affects thinking/behaviour and bodily responses				
	TAU	132	55	−1.45 (−1.87, −1.03)p<0.001
		3.62 (1.56)	3.84 (1.29)	
	GSH-CBT	133	63	
		3.85 (1.52)	5.43 (1.17)	
Ability to notice negative thoughts				
	TAU	132	56	−1.42 (−1.90, −0.95)p<0.001
		3.86 (1.46)	4.04 (1.33)	
	GSH-CBT	133	63	
		4.20 (1.47)	5.44 (1.25)	
Ability to challenge negative thoughts and seek to have more helpful thoughts				
	TAU	133	56	−2.04 (−2.48, −1.60)p<0.001
		2.73 (1.39)	3.02 (1.36)	
	GSH-CBT	133	62	
		2.99 (1.47)	5.10 (1.17)	

Our primary outcome is the BDI-II score. We used a reduction in score from baseline to 4 months as a measure of treatment response [18]. At both 4 and 12 months there were also significantly higher proportions of participants achieving a 50% reduction in BDI-II score in the GSH-CBT arm. At 4 months 43/101 (42.6%) participants in the GSH-CBT arm achieved this reduction compared to 25/102 (24.5%) at 4 months (odds ratio 2.28, 1.25 to 4.17, p = 0.008) in TAU. Recovery at 12 months was 31/62 (50.0%) for GSH-CBT, and 20/55 (36.4%) for TAU (odds ratio 1.75, 0.83 to 3.70, p = 0.14).

### Dose response

Overall, 3/16 (18.8%) recovered when they attended one or fewer self-help support consultations, compared with 40/85 (47.1%) in those attending 2 or more consultations (odds ratio 3.85 (95%CI 1.01–14.7 p<0.049)).

Multiple aspects of mental health literacy were significantly improved in the intervention group (Table 4). Scores on the Client Satisfaction Questionnaire were also higher in the GSH-CBT group at one month, with a mean (SD) of 27.6 (4.4) compared to 20.0 (5.4) in TAU; a mean (95% CI) difference of 7.6 (5.7 to 9.5), p<0.001, based on a two sample t-test.

Since the between group effects always depends on the type of control group used, within effects are reported in Table 5.

**Table 5:** Within-group changes BDI-II for completers and ITT analysis at 4 and 12 months.

Outcome	Population	Time Point	TAU		SH-CBT	
			Mean Change from Baseline	95% CI	Mean Change from Baseline	95% CI
BDI-II	Completers	4 Months	−7.00	(−9.02, −4.98)	−12.14	(−14.04, −10.24)
BDI-II	Completers	12 Months	−8.00	(−11.71, −4.29)	−13.35	(−15.97, −10.74)
BDI-II	ITT	4 Months	−5.10	(−6.66, −3.54)	−8.70	(−10.33, −7.06)
BDI-II	ITT	12 Months	−3.14	(−4.72, −1.57)	−5.87	(−7.46, −4.29)

## Discussion

### Key points

The study successfully achieved its required sample size. The sample reflected a notable frequency of severe depression on BDI-II scores but the cohort was also well treated with pharmacotherapies for depression. There were significant gains at 4 months in terms of depression (BDI-II), and CORE total score.

The treatment is highly acceptable to participants and there is clear evidence of reduced clinical deterioration in mood for those receiving GSH-CBT. Importantly there were gains across several key outcome measures rather than in just one or two areas. We also observed significant gains in mental health literacy as well as in the CORE total score.

At 12 months, the benefits of self-help over TAU were maintained on the BDI-II.

*Strengths*: This study is community-based and reflects “real-life” referrals from a range of urban and semi-urban family practices with varying levels of socioeconomic deprivation. The pragmatic approach to recruitment is easily reproducible in clinical practice. We produced the largest RCT to date examining guided CBT written self-help. The recruitment model proved successful – overcoming an issue of problematic recruitment in a series of other studies in this field. The intervention is quickly delivered and is clinically effective. Overall 50.4% of people completed 3 or 4 sessions over the month's treatment.

Data collection, entry and analysis were performed independently to ensure the result was free from the potential bias induced by participation of the author of the materials in the study team. Furthermore the initial training of the guided self-help support workers was delivered by another member of staff. Remote randomisation and collection of data by a worker independent of the person delivering treatment also minimised bias.

### Weaknesses

Our follow-up rate of 72.2% at 4 months is better than we predicted but is poorer at 12 months (41.6%) than we had hoped. We lack data on those participants who proved uncontactable and no data analysis is possible for those people. In the GSH-CBT arm, around 50% of people attended 2 or fewer sessions and 22% failed to take up the treatment.

Although the study design was analysed blind, because only one research assistant recruited and followed up patients it was not possible for the RA to retain blindness. Our use of self-rated questionnaires will mitigate against bias. Another area we would modify in future research would be the exclusion of people with low energy, concentration difficulty and tiredness. The rationale was to focus on people who could use the materials – but this excluded a small number of people with some “core” symptoms of depression. GP access to treatment as usual was available in both arms and could include a wide variety of possible interventions including medication, psychology, counselling and psychiatry referral. We hope that the randomisation process will have equalised out the support needed in participants in the two arms. Our future economic analysis will allow a better description of these additional inputs in each arm.

The study design did not control for the impact of the relatively low level of human supportive contact in the GSH-CBT arm. The study fulfils criteria for minimal contact self-help with a maximum of 160 minutes of support and mean of 124.3 minutes of support. It is not known whether this provides optimal benefit, and whether improved or equal outcomes could be achieved with either shorter or longer, or fewer or more guided support sessions. We also do not know the relative contributions of the book, the support or a combination of both. Other support options are also available including telephone based support. We chose face to face support delivered in the patient's own general practice in order to build the service into existing ways of working. These issues of type, place, content and extent of support are areas that could be the focus of future studies.

### Conclusions

The results provide strong evidence that the GSH-CBT package is effective when offered as a combination of the book plus up to 4 face to face support sessions. This is in line with other research confirming the importance of guidance in improving the impact of bibliotherapy [4]. In the current study, at 4 months, showed 42.6% recovered in the GSH-CBT group and 24.5% in the TAU group and an odds ratio for recovery of 2.28. The interaction between factors that predict improvement to GSH-CBT will be explored in a further paper.

### Future research

A replication study in other settings should focus on providing shorter support sessions as the Gellatly et al review suggests that all that is required is supportive monitoring [4]. A desirable next step would be to repeat the study comparing GSH-CBT directly with antidepressant medication.

## Supporting Information

Checklist S1 **Consort 2010 checklist of information to include when reporting a randomised controlled trial.**
(PDF)

Protocol S1 **Trial Protocol.**
(PDF)

Figure S1 **Consort Diagram.**
(TIF)
