# Correction: Guided Self-Help Cognitive Behavioural Therapy for Depression in Primary Care: A Randomised Controlled Trial

**DOI:** 10.1371/annotation/998d1a71-7c06-4ebd-8deb-d5db5ad21c31

**Published:** 2013-09-17

**Authors:** Christopher Williams, Philip Wilson, Jill Morrison, Alex McMahon, Andrew Walker, Lesley Allan, Alex McConnachie, Yvonne McNeill, Louise Tansey

The fifth author's first and last names were switched. The correct name is: Andrew Walker. The correct citation is: Williams C, Wilson P, Morrison J, McMahon A, Walker A, et al. (2013) Guided Self-Help Cognitive Behavioural Therapy for Depression in Primary Care: A Randomised Controlled Trial. PLoS ONE 8(1): e52735. doi:10.1371/journal.pone.0052735

The last sentence in the Results section was incorrectly stated. The sentence should read: All recordings examined were rated satisfactory against a brief adherence checklist. 

