# Supplementary material for: Guided Self-Help Cognitive Behavioural Therapy for Depression in Primary Care: A Randomised Controlled Trial
Source: PLoS One. 2013 Jan 11;8(1):e52735. doi: 10.1371/journal.pone.0052735 (PMC3543408; doi:10.1371/journal.pone.0052735)

Protocol

An evaluation of the effectiveness of structured Cognitive Behaviour Therapy self-help materials delivered by a self-help support worker within Primary care.

Aims: The primary aim is to investigate the clinical effectiveness of '*Overcoming Depression'* in the treatment of depression when offered using a "2+1" model. Effectiveness will be measured using standardised outcome measures that address a broad range of outcomes. We will address:
1). Is a self-help CBT package delivered by a self-help support worker:
a). More effective than treatment-as-usual, as measured by recovery from depression, social functioning and service use?
b). More cost effective than treatment-as-usual?
2). Is the CBT self-help package acceptable to patients experiencing depression and also to their practitioners?
3). Does use of the self-help package enhance patient knowledge of the causes and treatment of depression?

Research questions: The hypotheses are:
1). Patients using the CBT self-help materials will have
a) improved mood measured on the Beck Depression Inventory (BDI-II)
b) improved social functioning measured on the Clinical Outcome Measure in Routine evaluation - Outcome Measure (CORE-OM).
c). lower health care costs
d). improved knowledge of the causes and treatment of depression compared to the control group receiving treatment as usual.
2). Written self-help will be acceptable to both patients and staff within a primary care setting.

Plan, methods, expertise available, statistical power: A randomised-controlled trial will compare the CBT self-help treatment package with treatment as usual. Patients who present with symptoms of depression, have a BDI-II score of 14 or more, are aged eighteen or above and are able to use the materials (i.e. have no visual or reading problems, learning difficulties or dementia) will be offered entry into the study. Patients with suicidal intent (score of 2 or more on the BDI-II suicidal thoughts item) and impaired concentration and motivation (as measured by a score of 7 or more on the combined BDI II items for energy (item 15, concentration difficulty (item 19)and tiredness (item 20)) will be excluded.

Any member of the primary care team including GPs, practice or district nurses and health visitors will be able to refer to the study, thus we shall be providing a resource for the whole team. There are many potential benefits of using self-help approaches, however self-help is not for everyone therefore the process of initial recruitment is important. Recruited patients will need to be willing and able to use the materials and their use should be clinically appropriate. A simple recruitment procedure will be used where any apparently suitable patient can be immediately booked in on the Practice Computer booking system to see the study Research Assistant (RA), alternatively their details are left for the RA who then contacts them via letter to invite them for an appointment.

The RA will contact the patient by phone before the initial appointment to confirm that they have received the Patient Information sheet, answer any questions about the study and confirm whether they wish to attend the appointment to find out more.

At the initial appointment the RA will provide information about the study and after obtaining written informed consent will complete the BDI-II, apply the inclusion/exclusion criteria and gather baseline assessment measures.

The number of people with depressive symptoms offered and not offered the approach by the primary health care practitioner and the rates of acceptance to see the RA will also be anonymously recorded. The number of people who decline entry into the study or fail to meet the inclusion criteria will be anonymously recorded by the RA, as will their age, gender and postcode. Those who do not meet the inclusion criteria because of suicidal ideation or poor energy / motivation levels will be given a booklet which contains educational information about depression and sources and types of help which are available (other than the study).

Patients will be allocated by the RA at random to treatment as usual (TAU - a standardised letter is sent to the GP about this decision) or to the self-help arm (- the same standardised letter is sent to the referring practitioner and in addition an appointment made with the SHSW). The standardised letter contains information on whether the patient attended the appointment and if so, when they were seen, whether they consented to enter the study, whether they were eligible for the study, which arm of the study they were allocated to and their score on the BDI-II suicide screening question.

Randomisation will be by a central telephone randomisation service run by the Robertson Centre for Biostatistics, University of Glasgow. The randomisation will be by blocks and be stratified by GP practice. Patients in both treatment groups will be assured that they can withdraw from the study at any time without their future care being affected.

Expertise available: The applicants provide experience and skills in using and evaluating self-help materials (CW, GW), running large randomised controlled studies in primary care (JM, PW, CW, IW) and in economic and statistical evaluation (AM, AW).

Statistical advice: The sample size in this study is based on the effect found for a comparison between cognitive behaviour therapy and usual care using the Beck inventory score at 4 months as the principal outcome (Ward et al, 2000). This study was selected as it used a similar setting/patient population and inclusion/exclusion criteria as our study including a GP treatment as usual group. This is not otherwise available for the existing bibliotherapy studies. Ward et al's study found an effect size of 0.43 favouring CBT. This reflects an improvement of 4.5 points on the Beck Depression Inventory – a clinically useful gain. This study experienced a drop out rate of 10% by the 4 month follow up and this corresponds to the 0-10% drop-out identified by Cuijpers (1997). Our intervention may be expected to have an efficacy that will be similar to, or perhaps slightly

lower than was found in the Ward et al study. We have also identified a higher drop-out rate in our pilot study of around 33% - and this is equivalent across both arms. With 300 patients randomised in equal proportion to usual treatment or self-help, then using a two sample two-sided t-test the study will have 85% power to detect a difference in Beck Inventory Score of 0.43 standard deviations, assuming a drop out rate of 33%. The study would have 80% power to detect an effect size of 0.40 standard deviations, and if the drop out rate (loss to follow up) was nil, the study would have 80% power to detect an effect size of 0.33 standard deviations of Beck Inventory. We expect the common standard deviation for Beck Inventory to be around 10 units, so we would be able to detect a mean difference of around 4 units. These calculations assume a drop-out rate of 33% between randomisation and follow-up as suggested by our pilot study. We estimate that we will need to access 5-7 practices within Glasgow, and to continue to recruit at a rate of 3-4 patients a month per practice, in order to achieve recruitment over a 12 month period. The pilot confirms that this is attainable.

Retention of patients once recruited is important. If informed written consent is obtained, the first appointment with the SHSW will focus on assessment and appropriate use of the self-help materials. The first workbook will be introduced and section 1 covered including information on how to get the most out of the materials. This first workbook helps identify which additional workbooks from the course might be helpful. A second appointment time will be agreed and a copy of workbook 1 (clinical assessment) will be given to the patient to take away and read between the sessions. At session 2, a review of the first workbook will occur to allow clarification of what has been learned, and how to put what has been learned into practice. A decision is then made about which problem areas to focus upon and to identify the additional 1-2 treatment workbooks that will be used between sessions 2 and 3. At session 3, a final review of progress will be offered. The relapse prevention workbook and up to one or two additional workbooks will be offered at this final appointment. At each appointment also a brief risk assessment is completed using the combined “risk” questions from the CORE-OM and BDI questions. At the end of each appointment the SW gives the patient the relevant measures and leaves them to complete them, the participant then seals them in an envelope so that they can be returned to the RA without the SW seeing them.

In the TAU arm, the follow-up evaluations are completed either by post or face-to-face over a 10-15 minute period at weeks 2 and 4. The aim is to provide additional face-to-face contact to those in the TAU arm, to reduce the possibility that any clinical improvement in the self-help arm is due to non-specific “support” factors. The four and twelve month assessment will be either face to face or sent by post for both arms of the study.
In both arms, subjects are asked to complete a BDI-II, EQ-5D, CORE, PQ2 and CSQ (where appropriate). Patients who fail to attend an appointment will be contacted by phone and letter and offered a further appointment/sent out the evaluation questionnaires. At any time during treatment, the patient in the self-help condition can contact the SHSW to ask questions about the use of the workbooks and the frequency, length and content of such contacts will be recorded. Patients can also arrange to see their doctor or other health care

practitioner as normal. The intervention by the self-help support worker is only to support the use of the self-help materials and they will not offer “advice” separate from the intervention.
The measures used in the study fall into several categories:

- Beck Depression Inventory-II (BDI-II: Beck et al., 1996). 21 item assessment for depression.
- CORE-OM (Clinical Outcome Measure in Routine evaluation - Outcome Measure, CORE System Group, 1998). Comprising 34 items, the instrument measures four domains: subjective well-being, symptoms, life-functioning, and risk to self/others.

Economic measures:

- Euroqol (EQ-5D; Brooks, 1996). A brief 7 item measure of health status, health related quality of life, and lifestyle which has been developed for use in evaluating health and the outcomes of heath care.

The economic evaluation will focus on net economic costs to the NHS and health benefits to patients. For both arms of the trial patient notes held by the GP practice will be used to identify (i) the number of contacts with the primary care team (including which member of staff, which setting), (ii) drugs prescribed, (iii) other services received (e.g. community mental health team contacts), (iv) admissions. All contacts with the self-help support worker and other services will be recorded. The focus will be on collecting resource use data that are related to the person's depression. The resources used will be valued from standard sources (BNF for drugs, "Scottish Health Service Costs" for hospital and community services, PSSRU data for primary care contacts). These data will be used to calculate the difference between the two arms on an intention-to-treat basis. Health benefits will be measured and valued using EQ5D to estimate gains in quality-of-life. The results will be expressed as a net cost per QALY gained to the end of trial follow-up. Specific consent will be requested from the patient to allow a review of the GP records to confirm this information.

Other measures include:

- Adherence and competency criteria will monitor standards of delivery by the self-help support worker. A selection of sessions will be taped (where patient consent is obtained) and rated for adherence to the delivery model.
- Patient use of the workbooks, satisfaction and knowledge (PQ; based upon Whitfield et al 2001), and satisfaction with the service delivered using the Client Satisfaction Questionnaire (CSQ Larsen et al, 1979).
- The length and severity of depression will be recorded to give a measure of the extent and chronicity of depression. The severity of depression will be recorded by the BDI-II score. The length of depression will be recorded at initial recruitment and in addition the presence of other confounders recorded at that time (use of antidepressants, use of other self-help materials).
- An approximation of their reading ability.
- Whether and to what extent they self-medicate with alcohol or drugs

Administration of measures: Baseline, one, four and 12-month assessments for both arms will be completed face to face in the practice or at home or by post/telephone or email.

. The TAU arm will receive the same measurements sent as per the timescale illustrated below. The researcher will be aware of which arm patients are in, however all data entry and analysis will be completed blind. In addition, the use of objectively rated self-completion questionnaires will minimise any risk of observer bias. The independent and blinded analysis by AM (Robertson Centre) will also minimise any bias in the evaluation.

Timing	At recruitment Week 0 by RA	At 1st and 2nd session/ Week 2 by RA	At final session (1 month) by RA	3 months post treatment (4 months) by RA	11 months post treatment (12 months)
Arm 1 **Usual Treatment**	BDI-II, CORE EQ-5D, PQ1	BDI-II CORE	BDI-II, CORE EQ-5D, PQ2 CSQ	BDI-II CORE, EQ-5D Economic evaluation	BDI-II, CORE, EQ5D, Economic evaluation
Arm 2. **"2+1" supported self-help**	BDI-II, CORE EQ-5D, PQ1	BDI-II CORE	BDI-II, CORE EQ-5D, PQ2 CSQ	BDI-II CORE, EQ-5D Economic evaluation	BDI-II, CORE, EQ5D, Economic evaluation

The primary outcome will be a comparison between the BDI-II scores for the two randomised groups (self help plus treatment as usual versus TAU) using a 2 sample 2-sided t-test. Further analyses that adjust the treatment effect for a pre-specified set of baseline covariates thought to be of influence on the treatment effect such as use of antidepressants, other self-help materials, and the chronicity of depression using Normal Linear models, will be considered. Secondary analyses will examine the impact of treatment on the four main CORE domains (well-being, symptoms, life functioning and risk to self/others), subjective use of alcohol and drugs, changes in use of anti-depressants and also patient knowledge, and the cost implications of use, and patient and practitioner perspectives of the effectiveness and acceptability of this treatment approach. The approach by Jacobson et al, (1991) to present change in the group under study at the level of the individual will also be used. Categorical data will be compared between the two groups using chi-squared tests and logistic regression to adjust for covariates.

7). Timetable of work.
If the study is funded, we intend to continue to employ our current RA and SHSW.

- April 2004: Continue to recruit from the existing five practices
- April-March 2005: establish adequate recruitment. If required increase to recruiting from 7 practices (2 additional practices have been identified). Recruitment/follow-up during this period.
- April 2005-March 2006 Patient follow-up.
- April 2006-October 2006: RA and an additional part-time RA will gather the economic data from Practices, code and submit data for final analysis. Final report.

13). Relevant additional materials. (e.g. questionnaires etc)

Using self-help materials.

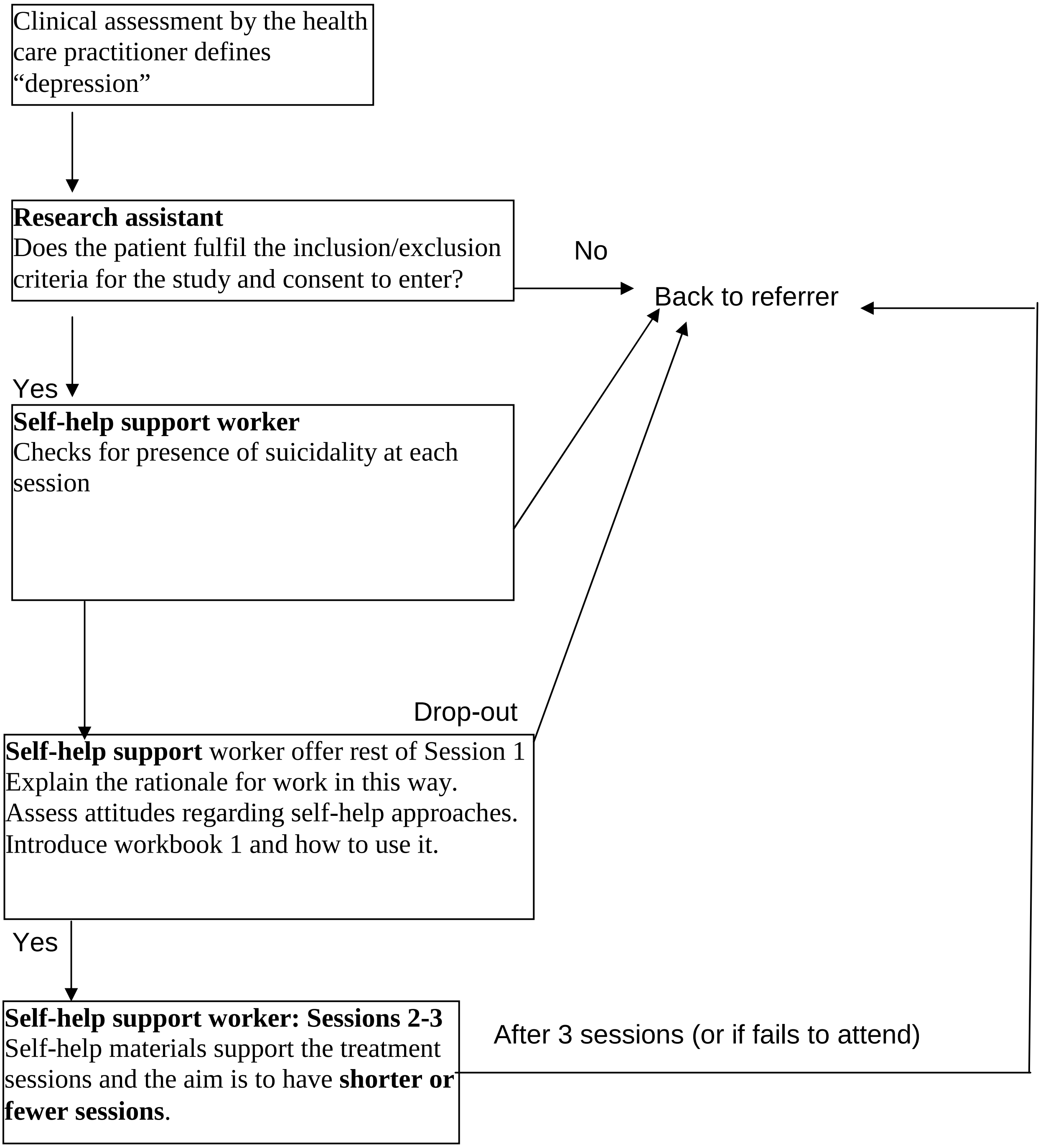

Supplement: Protocol S1 — Trial Protocol. (PDF) [file pone.0052735.s002.pdf]
