# Supplementary figures and images for: Guided Self-Help Cognitive Behavioural Therapy for Depression in Primary Care: A Randomised Controlled Trial
Source: PLoS One. 2013 Jan 11;8(1):e52735. doi: 10.1371/journal.pone.0052735 (PMC3543408; doi:10.1371/journal.pone.0052735)

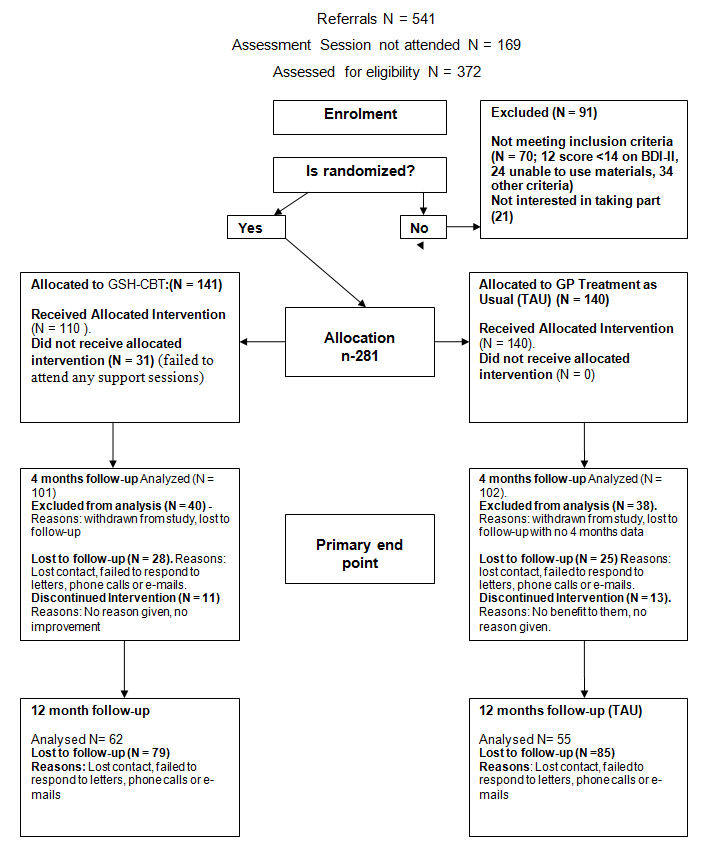

Supplement: Figure S1 — Consort Diagram. (TIF) [file pone.0052735.s003.tif]
